# The anti-addictive drug ibogaine modulates voltage-gated ion channels and may trigger cardiac arrhythmias

**DOI:** 10.1186/1471-2210-11-S2-A1

**Published:** 2011-09-05

**Authors:** Michael Kovar, Xaver Koenig, Ágnes Mike, René Cervenka, Péter Lukács, Hannes Todt, Walter Sandtner, Karlheinz Hilber

**Affiliations:** 1 Department of Neurophysiology and Neuropharmacology, Center for Physiology and Pharmacology, Medical University of Vienna, 1090 Vienna, Austria

## Background

Ibogaine is an alkaloid derived from the African shrub *Tabernanthe iboga*. Psychoactive properties of ibogaine have been known for decades, but more recently the drug has received much attention because of its promising “anti-addictive” actions. Thus, ibogaine and its derivatives are being studied as potential treatment for opioid and stimulant abuse, as well as for alcoholism and smoking. Because ibogaine has a complex pharmacology and is known to interact with numerous different cellular targets, its potential to generate adverse effects is significant. Besides the expected neurotoxic actions, ibogaine may e.g. also affect the heart. Thus, several cases of sudden death after ibogaine use were reported, which have been hypothesised to be related to cardiac arrhythmias. In accordance, a severely prolonged QT interval of the electrocardiogram and ventricular tachyarrhythmias were observed in a woman after she had taken ibogaine.

## Methods

To study possible mechanisms by which ibogaine may trigger cardiac arrhythmias, we explored ibogaine’s effects on the function of cardiac voltage-gated ion channels, by using the whole-cell patch-clamp technique. In addition, we also tested the ibogaine derivative 18-methoxycoronaridine (18-MC), which is considered less toxic.

## Results

We found that currents through human ERG (hERG) potassium channels, heterologously expressed in tsA201 cells, were inhibited by ibogaine in low micromolar concentrations (IC_50_: 3 µM). In addition, ibogaine significantly altered the hERG channel gating properties. The IC_50_ of hERG current inhibition by 18-MC was 15 µM. Heterologously expressed human Na_V_1.5 sodium channels were also affected by ibogaine. For sodium current inhibition about 25-fold higher ibogaine concentrations were needed than for hERG. Finally, experiments on isolated adult mouse cardiomyocytes showed that ibogaine also affects currents through voltage-gated ion channels in their native environment.

## Conclusions

Because the ibogaine concentrations in animal and human plasma after ibogaine uptake reach low micromolar concentrations which impair the function of cardiac ion channels, the drug must be considered a potential cardiac arrhythmia risk.

